# Mexican Patients With Suspected 22q11.2 Deletion Syndrome: Clinical Characterization and Molecular Findings by Fluorescence In Situ Hybridization and Multiplex Ligation‐Dependent Probe Amplification

**DOI:** 10.1002/mgg3.70153

**Published:** 2025-10-30

**Authors:** Thania Alejandra Aguayo‐Orozco, Horacio Rivera, Luis E. Figuera, Eduardo Esparza‐García, Francisco Javier Perea‐Díaz, Ana Rebeca Jaloma‐Cruz, Lourdes del Carmen Rizo‐de la Torre, Ma. Guadalupe Domínguez‐Quezada

**Affiliations:** ^1^ División de Genética Centro de Investigación Biomédica de Occidente, Instituto Mexicano del Seguro Social Guadalajara Jalisco México; ^2^ Doctorado en Genética Humana Centro Universitario de Ciencias de la Salud ‐ Universidad de Guadalajara Guadalajara Jalisco México; ^3^ Unidad Médica de Alta Especialidad, Hospital de Pediatría ‐ Centro Médico Nacional de Occidente Instituto Mexicano del Seguro Social Guadalajara Jalisco México; ^4^ División de Medicina Molecular Centro de Investigación Biomédica de Occidente, Instituto Mexicano del Seguro Social Guadalajara Jalisco México

**Keywords:** 22q11.2 deletion syndrome, fluorescence in situ hybridization, Mexican patients, multiplex ligation‐dependent probe amplification, phenotypic variability, *TBX1* gene

## Abstract

**Background:**

The 22q11.2 deletion syndrome (22q11.2DS) is mostly caused by deletions of 3 and 1.5 Mb, referred to as typical deletions, although atypical deletions have also been reported. The commonest features are congenital heart disease, immunodeficiency, facial dysmorphism, and developmental delay. However, phenotypic variability is remarkable, and the underlying mechanisms remain poorly understood.

**Objective:**

To determine copy number variations (CNVs) in the 22q11.2 region and their association with clinical manifestations in Mexican patients with suspected 22q11.2DS.

**Methods:**

Fluorescence in situ Hybridization (FISH) and Multiplex Ligation‐dependent Probe Amplification (MLPA) assays were performed in 80 patients with suspected 22q11.2DS. Clinical characterization was carried out according to the criteria used by the 22q11.2 Consortium.

**Results:**

FISH detected deletions in 51%, while MLPA detected CNVs in 54%. Typical deletions were observed in 86% of patients, whereas atypical deletions were found in 14%, including CNVs involving single genes (*TBX1, TOP3B*, and *PRODH*). Three families were identified with the 3 Mb deletion and exhibited a heterogeneous phenotype that cannot be explained by the microdeletion alone.

**Conclusion:**

22q11.2DS is a complex disorder for which MLPA is recommended to detect atypical deletions in FISH‐negative patients, and to define deletion size, breakpoints, and genes in FISH‐positive ones.

## Graphical abstract

Evaluation of 80 Mexican patients with suspected 22q11.2 deletion syndrome, including detailed phenotypic characterization. Multiplex ligation‐dependent probe amplification identified both typical and atypical deletions, underscoring the need for complementary approaches to fluorescence in situ hybridization. Neither the size nor the type of deletion fully account for the observed clinical variability.

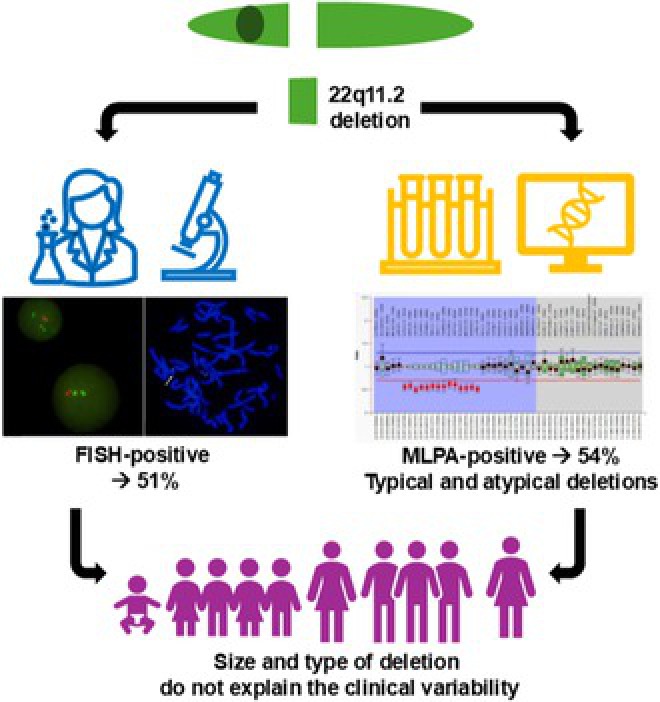

## 1 Introduction

The 22q11.2 microdeletion is the most common chromosomal loss in humans, with a frequency ranging from 1 in 1000 in miscarriage reports to 1 in 6000 in live births. Its prevalence in adults has increased in recent years due to improved child survival resulting from genetic screening and advances in pediatric care (Maisenbacher et al. 2017; Gavril et al. 2022; Purow et al. 2025). At present, the 22q11.2 deletion syndrome (22q11.2DS) encompasses several entities described before the molecular era, such as DiGeorge syndrome (DGS; OMIM #188400), velocardiofacial syndrome (VCFS; OMIM #192430), CATCH‐22, Takao syndrome, and others. These syndromes share key clinical features such as congenital heart disease (CHD), mainly conotruncal defects, soft palate abnormalities, immunodeficiency, facial dysmorphism, and psychomotor and developmental delay, as well as an increased risk of schizophrenia (McDonald‐McGinn et al. 2015; Cortés‐Martín et al. 2022). Despite the existence of a classical delineation of the major traits, the notable phenotypic variability continues to hinder the definition of a precise phenotype–genotype relationship and the elucidation of the underlying mechanisms.

In general, the 22q11.2 microdeletion occurs in heterozygosity and results from non‐allelic homologous recombination (NAHR), facilitated by eight blocks of low‐copy repeats (LCR), designated LCR22A to LCR22H, arranged in the centromere‐to‐telomere direction (Burnside 2015; Vervoort et al. 2025). These LCRs, also referred to as segmental duplications, share more than 96% sequence identity and therefore predispose to chromosomal rearrangements through unequal meiotic exchange, usually resulting in deletions or duplications (Morrow et al. 2018; Vervoort et al. 2025). The 22q11.2 microdeletion occurs de novo in approximately 90% of patients and is inherited from either parent in the remaining 10% (Cortés‐Martín et al. 2022; Purow et al. 2025). Deletions of 3 and 1.5 Mb are considered typical and account for most cases; the former spans LCR22A to LCR22D and represents around 90%, while the latter involves LCR22A to LCR22B and is observed in 5%–7%. The remaining 3%–5% correspond to atypical deletions that differ from typical ones in size and breakpoints and are not favored by LCRs (Koczkowska et al. 2017; Campbell et al. 2018; Burssed et al. 2022; Gavril et al. 2022). In the 22q11.2DS, as in other contiguous gene syndromes, haploinsufficiency of specific genes contributes to distinctive clinical features. For instance, the complex phenotype associated with abnormal pharyngeal development is largely attributable to the loss of the *TBX1* gene (OMIM *602054) (Ping et al. 2016; Purow et al. 2025).

In Mexico, fluorescence in situ hybridization (FISH) has been widely employed for the diagnosis of 22q11.2DS since the 1990s, despite relevant limitations inherent to the use of commercial probes such as N25, TUPLE1, or TBX1. These probes cannot distinguish typical from atypical deletions, fail to identify all missing genes within the LCR22A–LCR22B region, may not detect distal deletions, and do not provide sufficient information in complex cases involving multiple deletions or duplications within the 22q11.2 region (Evers et al. 2016). In Latin America, detection rates of 22q11.2 deletions vary widely, ranging from 20% in Brazilian patients with CHD and facial dysmorphism to over 50% in selected cohorts with conotruncal heart disease, highlighting both the influence of clinical criteria on diagnostic yield and the lack of data for Mexican patients (Cuturilo et al. 2016; Diniz et al. 2020; Ramírez‐Velazco et al. 2018). Although FISH continues to be used in most Mexican cytogenetic laboratories due to its affordability and the rapid turnaround of results, it should not be employed as the sole diagnostic method, particularly in patients in whom the deletion cannot be detected (Poirsier et al. 2016). Multiplex Ligation‐dependent Probe Amplification (MLPA) is designed to detect copy number variations (CNVs), including deletions and duplications of multiple genes, and is considered the gold standard molecular test for the diagnosis of genomic gain and loss disorders (Evers et al. 2016; Nouri et al. 2016; Gavril et al. 2022). The detection rate of MLPA in patient cohorts varies depending on the selection criteria. For instance, studies applying this technique to heterogeneous CHD cases reported rates between 3% and 20% (Bolunduț et al. 2024; Floriani et al. 2022); while another study considering two compatible clinical traits documented 23% (Rojnueangit et al. 2020). MLPA is particularly valuable for characterizing deletions: in patients previously identified by FISH, it determined the type and size of deletions in 100% of cases, and it also revealed atypical deletions in FISH‐negative patients (Cuturilo et al. 2016; Delea et al. 2022; Evers et al. 2016; Rojnueangit et al. 2020). Moreover, MLPA has identified intragenic *TBX1* deletions in patients with 22q11.2DS features but without typical deletion (Aguayo‐Gómez et al. 2015; Delea et al. 2022). Thus, it has been proposed as a first‐line test in syndromic heart disease due to its higher sensitivity and specificity than FISH and its lower cost compared with chromosomal microarray analysis (CMA). Emerging technologies take advantage of digital PCR and TaqMan‐based dosage analysis; however, despite their low cost per reaction, they do not provide accurate information regarding the type and size of deletions, as MLPA does (Floriani et al. 2022; Bolunduț et al. 2024; Ranaweera et al. 2025). In view of these advantages and limitations and given the remarkable phenotypic variability of 22q11.2DS together with the scarce research in the Mexican population, this study aimed to characterize CNVs within the 22q11.2 region and their possible association with clinical features in an underrepresented population.

## 2 Material and Methods

### 2.1 Ethical Compliance

The study was reviewed and approved by the National Commission for Scientific Research of the “Instituto Mexicano del Seguro Social” (IMSS) under registration number R‐2017‐785‐088. The commission adheres to internationally recognized ethical standards, including the Declaration of Helsinki and the International Ethical Guidelines for Health‐Related Research Involving Humans. Written informed consent was obtained from all participants or their parents, who also provided authorization for the publication of the findings.

### 2.2 Patients and Clinical Characterization

Between 2017 and 2023, a total of 80 patients were studied. Of these, 76 had a presumptive clinical diagnosis of 22q11.2DS, and the remaining four patients had been previously examined in the Ramírez‐Velazco (2014) project, with a confirmed diagnosis by FISH. Patients were included if they presented two or more major clinical features, according to the 22q11.2 Consortium criteria (McDonald‐McGinn and Sullivan 2011). All patients were clinically evaluated by an expert medical geneticist, who completed a standardized clinical form that also captured additional phenotypic features beyond the major, intermediate, and minor criteria. Family history of the parents and other relatives with suggestive traits was assessed to identify probable familial cases. Clinical characterization was updated at each subsequent visit to the genetics service, and electronic medical records were reviewed to complement the phenotypic description with evaluations performed by other medical specialists.

### 2.3 Karyotype and Fluorescence In Situ Hybridization

Chromosome preparations from lymphocytes were obtained according to standard laboratory procedures. Briefly, peripheral blood was cultured in RPMI 1640 medium supplemented with phytohemagglutinin for 72 h at 37°C. Colcemid, hypotonic KCl solution, and a fixative solution (methanol: acetic acid, 3:1) were added following established protocols. Karyotyping was performed on at least 16 metaphases, with a resolution of 550 bands. For the FISH technique, a dual probe was used according to the manufacturer's instructions (Cytocell). The probe included complementary sequences for the *HIRA* (*TUPLE1*) gene at 22q11.2 (orange spectrum) and for the *ARSA* gene at 22q13.3 (green spectrum) as a control. FISH analysis was conducted on at least 10 metaphases and 50 interphase nuclei. Results were interpreted and reported according to the recommendations of the *International System of Cytogenomic Nomenclature* (ISCN 2024) (Hastings et al. 2024).

### 2.4 Multiplex Ligation‐Dependent Probe Amplification Assays

DNA extraction was performed using the Qiagen Gentra Puregene kit, following the manufacturer's recommended protocol. DNA was quantified using a Nanodrop 2000. Two MLPA kits were used: SALSA P250‐DiGeorge lot B2‐0417 and SALSA P324‐22q11 lot B1‐1118 for the 22q11.2 region (MRC‐Holland, Amsterdam, The Netherlands). The first kit identifies the genes *IL17RA, SLC25A18, BID, MICAL3, USP18, CLTCL1, HIRA, CDC45, CLDN5, GP1BB, TBX1* (two probes), *TXNRD2, DGCR8, ZNF74, KLHL22, MED15, SNAP29, LZTR1, HIC2, PPIL2, TOP3B, RSPH14* (two probes), *GNAZ, RAB36, SMARCB1* (two probes), and *SNRPD3*; the second kit contains a mixture of specific probes for the genes *IL17RA, CECR2, SLC25A18, BID* (two probes), *MICAL3, USP18, PRODH, DGCR2, HIRA, CDC45, CLDN5, TBX1* (seven probes), *GNB1L, COMT* (two probes), *DGCR8, ZNF74, BCRP2, RIMBP3C, VPREB1, MIF*, and *SEZ6L*. Notably, the P250 kit contains complementary sequences for exons 2 and 7 of the *TBX1* gene, whereas the P324 kit includes seven probes corresponding to exons 1, 2, 3, 4, 7, and 8, as well as an additional probe located downstream. Additionally, the P250 and P324 kits together include probes for 1q32, 2q13, 4q35.1, 4q35.2, 5q31, 6q12, 7p15, 8p23.1, 8q12, 9q34.3, 10p12.31, 10p14, 11q22, 14q11, 17p11, 17p13.3, 20p12, and 22q13, providing a more comprehensive cytogenomic approach. All gene loci are based on the NCBI36/hg18 reference genome version.

## 3 Results

The patients were distributed into 71 probands and 9 relatives of the index cases. All patients underwent phenotypic characterization, FISH, and MLPA.

### 3.1 Karyotype

Regarding patients' sex, 49% (39/80) were registered as female and 51% (41/80) as male. Karyotype was obtained in 74/80 patients and was concordant with the sex assigned to each patient (46,XX or 46,XY). The absence of karyotype data in the remaining six patients was due to poor‐quality metaphases in the cell culture, and it was not possible to collect a new sample.

### 3.2 Fluorescence In Situ Hybridization (FISH)

All the patients (80/80) were studied by FISH on metaphase or interphase nuclei and were divided into two groups: *FISH‐positive*: 41 patients (51%) presented a deletion at 22q11.2. ISCN 2024 nomenclature: *ish del*(*22*)(*q11.2q11.2*)(*TUPLE1*−, *ARSA*+) for metaphase FISH, or *nuc ish*(*TUPLE1*) *× 1* for interphase FISH. *FISH‐negative*: 39 patients (49%) showed no deletion at 22q11.2 by FISH. ISCN 2024 nomenclature: *ish 22q11.2*(*TUPLE1*) *× 2* for metaphase FISH, or *nuc ish*(*TUPLE1*) *× 2* for interphase FISH.

### 3.3 Multiplex Ligation‐Dependent Probe Amplification (MLPA) Assays

**FIGURE 1:**
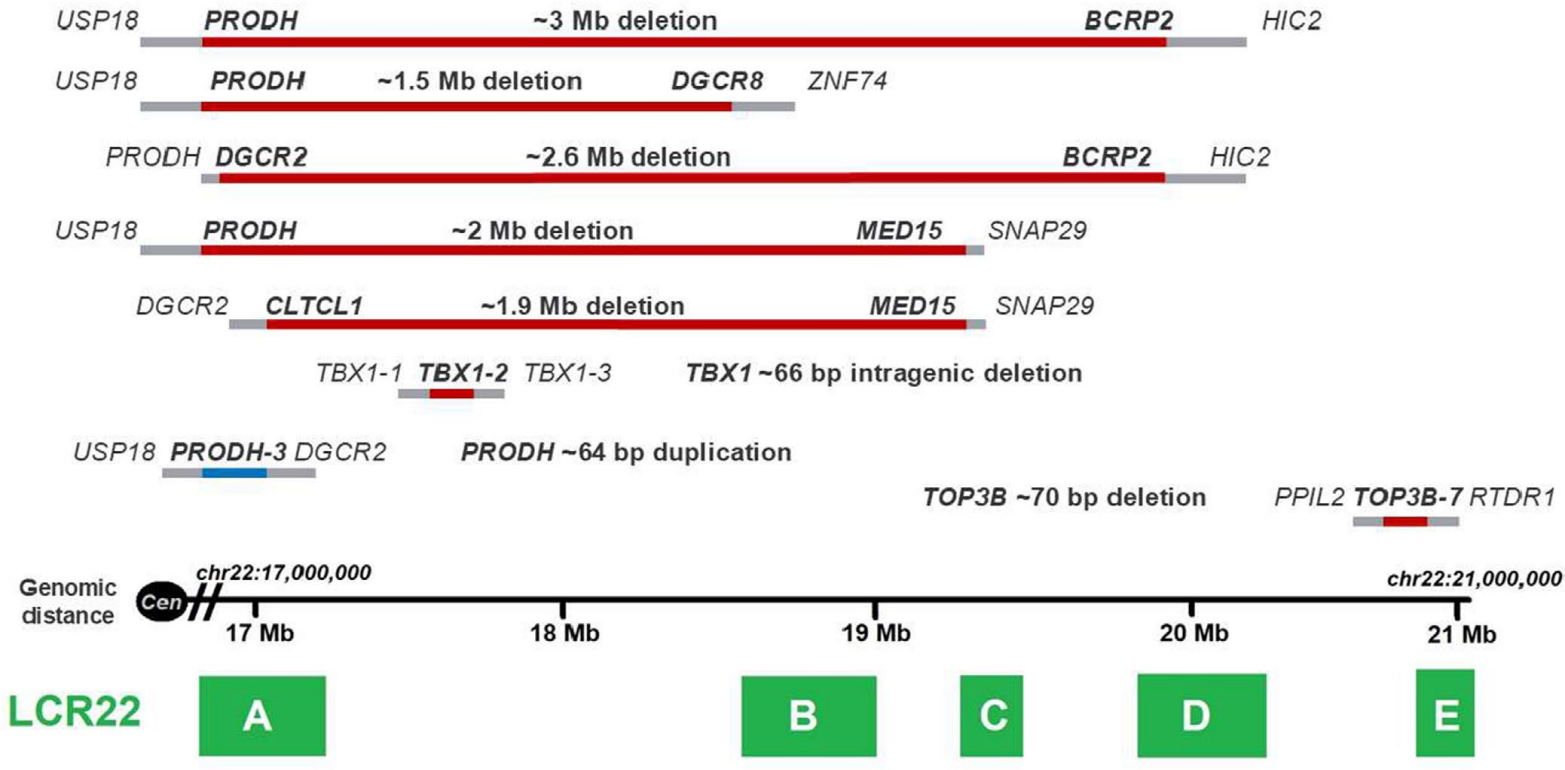
Copy number variations characterized by multiplex ligation‐dependent probe amplification. The approximate extent of the deletions is indicated by red lines, and the duplication of *PRODH* by a blue line. Positions are shown relative to the LCRs (in green boxes) at approximate genomic distances. Light grey lines represent the “grey areas”, defined as the regions between present and absent probes. Genes flanking the CNVs are shown in bold. bp, Base pairs; Cen, Centromere; LCR, Low copy repeats; Mb, Megabase. Genomic positions are based on the reference genome version NCBI36/hg18.

Eighty DNA samples were analyzed with MLPA (kits P250 and P324), which characterized the deletion previously detected by FISH in 41/80 patients (51%). In 2/80 FISH‐negative patients (3%), CNVs not detected by FISH were identified by MLPA: one patient had a loss of exon 2 of the *TBX1* gene, and the other showed a duplication of the *PRODH* gene (OMIM *606810). Thus, MLPA increased the detection rate to 54% (43/80). In the remaining 37/80 FISH‐negative patients (46%), MLPA disclosed no deletions or duplications. All results of MLPA analyses are presented in Table 1 and Figure 1. Detailed findings for each patient can be found in Data S1.

**TABLE 1:** General findings in 80 patients analyzed using karyotype, fluorescence in situ hybridization, and multiplex ligation‐dependent probe amplification.

QTY	KARYOTYPE	FISH	MLPA			
			CNVs	SIZE	TYPE	LCR localization
36	46,XX or 46,XY	POSITIVE	Deletion	~3 Mb	Typical	LCR22A‐D
1	46,XX	POSITIVE	Deletion Deletion	~3 Mb ~70 pb	Typical + *TOP3B*	LCR22A‐D LCR22D‐E
1	46,XY	POSITIVE	Deletion	~1.5 Mb	Typical	LCR22A‐B
1	46,XX	POSITIVE	Deletion	~2 Mb	Atypical	LCR22A‐C
1	46,XY	POSITIVE	Deletion	~1.9 Mb	Atypical	LCR22A‐C
1	46,XX	POSITIVE	Deletion	~2.6 Mb	Atypical	LCR22A‐D
1	46,XX	**NEGATIVE**	Deletion	~66 pb	Atypical *TBX1* (*exon 2*)	LCR22A‐B
1	46,XY	**NEGATIVE**	Duplication	~64 pb	Atypical *PRODH*	LCR22A+
37	46,XX or 46,XY	NEGATIVE	No CNVs	—	—	—
80 TOTAL						

*Note:* The two FISH‐negative patients with CNVs identified by MLPA are shown in bold.

Abbreviations: bp, base pairs; CNVs, copy number variations; FISH, fluorescence in situ hybridization; LCR, low copy repeats; Mb, megabase; MLPA, multiplex ligation‐dependent probe amplification; QTY, quantity.

### 3.4 Clinical Characterization

Remarkable phenotypic variability was observed in both MLPA‐positive and MLPA‐negative patients with CNVs at 22q11.2 (Table 2). The median age was 2 years in both groups. Across the evaluated criteria, different manifestations were observed. For example, cardiac anomalies included not only conotruncal defects but also valvular and septal defects, among others. The full spectrum of phenotypic variability for each criterion is presented in Data S2.

**TABLE 2:** Comparison of clinical manifestations in 80 patients classified by MLPA results.

Phenotypic feature	MLPA‐positive (CNVs 22q11.2) *n* = 43	MLPA‐negative (no CNVs) *n* = 37	*p*
**Major phenotypic features**			
Cardiac anomaly	32 (74.4%)	31 (83.8%)	0.307
Immune deficiency	**18 (41.9%)**	**7 (18.9.%)**	**0.027**
Palatal defects	12 (27.9%)	17 (45.9%)	0.094
Psychomotor delay or Intellectual disability	38 (88.4%)	31 (83.8%)	0.552
**Intermediate phenotypic features**			
Esophageal dysmotility	5 (11.6%)	6 (16.2%)	0.552
Hypocalcemia	16 (37.2%)	8 (21.6%)	0.129
Renal anomaly	3 (7%)	4 (10.8%)	0.545
Feeding and swallowing problems	17 (39.5%)	15 (40.5%)	0.927
Dental issues	6 (14.0%)	7 (18.9%)	0.548
Structural CNS anomaly	19 (44.2%)	17 (45.9%)	0.875
Spinal abnormality	4 (9.3)	7 (18.9%)	0.330
Ophthalmological/ophthalmic anomaly	7 (16.3%)	11 (29.7%)	0.151
Hearing loss	4 (9.3%)	9 (24.3%)	0.069
Behavioral or psychiatric problems	10 (23.3%)	10 (27.0%)	0.698
**Minor phenotypic features**			
Craniofacial dysmorphism	**43 (100%)**	**32 (86.5%)**	**0.013**
Postaxial polydactyly	3 (7.0%)	0 (0%)	0.245
**Other comparisons**			
Presence of other manifestations	31 (72.1%)	22 (59.5%)	0.233
Count of clinical findings according to study criteria	5.51 ± 1.81	5.65 ± 2.07	0.753

*Note:* Qualitative variables are expressed as frequencies (%). The *p‐*value for the count of clinical findings was calculated using Student's *t*‐test, while the Chi‐square test was applied for qualitative variables. Fisher's exact test was used for frequencies < 5. Statistically significant *p‐*values are shown in bold.

Abbreviation: MLPA, multiplex ligation‐dependent probe amplification.

It is important to highlight certain clinical features that were observed with some frequency but are not included in the Consortium criteria. These additional features include thrombocytopenia, cryptorchidism, inguinal and umbilical hernias, malnutrition, intestinal constipation, and varus foot, the latter observed in a familial case.

## 4 Discussion

The detection rate of 22q11.2 deletions in our patients was 51% by FISH, higher than the 42.1% reported in another case series in which patients were recruited based on at least two major criteria (Cuturilo et al. 2016), and markedly higher than the 17.9% detection rate in patients with only one major feature of 22q11.2DS (Cuturilo et al. 2016; Monteiro et al. 2013). A study in Brazil identified 20% of patients with 22q11.2 deletions among children with CHD and facial dysmorphism (Diniz et al. 2020), underscoring the need for a more precise diagnostic clinical algorithm to increase the detection rates (Monteiro et al. 2013). In 2018, our group found that 56% of patients with at least one major feature tested by FISH had the deletion; however, this likely reflects that almost all patients had conotruncal heart disease (Ramírez‐Velazco et al. 2018).

It is important to note that although the agreement between FISH and MLPA was high (97.5%), MLPA was able to characterize the size of the deletion in FISH‐positive patients and even detected an additional CNV in one of them. More importantly, two FISH‐negative patients were reclassified as MLPA‐positive CNV cases.

In this study, the P250 MLPA kit detected typical 3 Mb deletions (LCR22A‐LCR22D), typical 1.5 Mb deletions (LCR22A‐LCR22B), atypical deletions of approximately 1.9 and 2 Mb (LCR22A‐LCR22C), as well as a deletion involving the *TOP3B* gene (OMIM *603582). The high concordance between the results obtained with the P250 and P324 kits is largely due to the significant proportion of shared probes and their positions relative to the LCRs. Furthermore, combining the results from both kits enabled a more precise determination of distal and proximal breakpoints. However, an atypical ~2.6 Mb deletion (LCR22A‐D) and a *PRODH* duplication were detected exclusively with the P324 kit, as it includes probes that are not present in the P250 kit. Notably, the P324 kit also identified *PRODH* as the first gene lost in typical deletions, a relevant finding given that a recombination hotspot (LCR22A+) is near this gene (Guo et al. 2018).

The determination of CNV breakpoints allowed their classification and comparison with other case series. The largest study reported to date, which included 1421 patients with the 22q11.2DS, described the frequencies of CNV types according to their breakpoints, findings that are comparable to those observed in our cohort (Campbell et al. 2018). Typical 3 Mb deletions (LCR22A‐D) accounted for approximately 84% in both studies; 1.5 Mb deletions (LCR22A‐B) represented 5% and 2.3%, respectively, while atypical deletions were 11% and 14%. A more recent study of 59 patients reported 76% LCR22A‐D deletions, 3.3% LCR22A‐B deletions, and 20% atypical deletions, differences attributed to sample size (Gavril et al. 2022).

### 4.1 Genotype–Phenotype Observations

This study confirmed the broad clinical variability of the syndrome. Most patients with the typical 3 Mb deletion presented CHD and psychomotor delay as major features, along with swallowing difficulties and hypocalcemia as intermediate manifestations. Facial dysmorphism was observed in all patients, and notably, the three patients with polydactyly also carried this type of deletion. Familial cases, all carrying a 3 Mb deletion, showed both intra‐ and inter‐familial phenotypic variability. Cytogenetic screening of parents is essential, particularly when clinical features are suggestive. In fact, approximately 7%–10% of individuals with a 22q11.2 deletion have inherited the alteration from a parent, who usually presents with an attenuated or mild phenotype (McGinn et al. 2022).

The patient with a 3 Mb deletion combined with a *TOP3B* gene deletion (ID 18) exhibited a complex and severe phenotype, including CHD (pulmonary atresia, ventricular septal defect, and overriding aorta), recurrent infections, cleft palate, psychomotor delay, hypocalcemia, swallowing problems, and facial dysmorphism. Isolated *TOP3B* copy number changes have been reported in patients with syndromic features, suggesting that loss of this gene, in addition to the classical 3 Mb deletion, contributes to the severity of the phenotype in this patient (Daghsni et al. 2018; Riley et al. 2020).

The patient with an intragenic *TBX1* deletion (ID 48) presented pulmonary stenosis, primary immunodeficiency, moderate intellectual disability, hypocalcemia, microcephaly, hypermetropia, emotional lability, and facial dysmorphism. This aligns with previous reports indicating that deletions or pathogenic variants in *TBX1* alone are sufficient to produce a 22q11.2DS phenotype (Ping et al. 2016; Delea et al. 2022). However, genome‐wide techniques are required to fully characterize the mutational spectrum, while also considering potential epistatic pathogenic variants and environmental factors that may modulate the phenotype (Karbarz 2020; Pastor et al. 2020).

The monoallelic *PRODH* gain identified in a FISH‐ and MLPA‐negative patient (ID 69) who presented velopharyngeal insufficiency, short stature, psychomotor delay, swallowing difficulties, microcephaly, recurrent otitis media, and facial dysmorphism was considered an incidental finding. Heterozygous pathogenic variants in *PRODH* have been associated with an increased risk of schizophrenia (Kolar et al. 2023), and proximal deletions excluding *TBX1* but showing syndromic features (particularly neurological ones) have suggested candidate genes such as *DGCR6* (OMIM *601279) and *PRODH* (Carli et al. 2021; Zamariolli et al. 2023). In this patient, *PRODH* duplication may explain the psychomotor delay through neurological impairment; however, it does not account for the broader spectrum of 22q11.2DS manifestations.

The remaining CNVs identified in this study were smaller than 3 Mb, proximal, and included the *TBX1* gene, underscoring its key role irrespective of the size or specific gene content of each deletion (Purow et al. 2025).

When comparing clinical manifestations between MLPA‐positive and negative patients, only two variables showed significant differences: immunologic disorders and facial dysmorphism. Immunologic phenotypes including primary immunodeficiency (DiGeorge syndrome), thymic hypoplasia or agenesis, lymphopenia, sepsis, pneumonia, and recurrent infections were more frequent in MLPA‐positive patients (41.9%). This proportion is lower than the 77% reported by the 22q11.2DS Consortium and other studies, which range from 67% to 80% (McDonald‐McGinn and Sullivan 2011; Purow et al. 2025). This discrepancy likely reflects that patients without severe immunodeficiency in our cohort underwent immunological evaluation only after a 22q11.2DS diagnosis, leading to mild defects being overlooked at early stages. Nevertheless, our findings are consistent with previous reports noting that recurrent upper respiratory tract infections are common (Sullivan 2018; Lee et al. 2023). The observation that all MLPA‐positive patients displayed suggestive facial dysmorphism supports the notion that characteristic facies, including hooded eyelids and a bulbous nose tip, increases the probability of detecting the deletion (Farrera et al. 2019). Overall, these results highlight the marked clinical complexity of a syndrome associated with more than 180 phenotypic traits, underscoring the difficulty of establishing a precise genotype–phenotype correlation (Karbarz 2020).

Other clinical manifestations not included in the classical criteria were observed in both MLPA‐negative and ‐positive patients. Varus foot was notable, occurring in 4/41 (5%) of positive patients, a proportion higher than the 3.3% reported in a 2018 study, which found this alteration to be 30 times more prevalent in 22q11.2DS patients than in the general population (Homans et al. 2018). This finding supports the association, particularly as varus foot was observed in a familial case.

Some intermediate and minor features have been suggested to warrant classification as major traits (Monteiro et al. 2013). Accordingly, characterization of the phenotype in our cohort may inform the development of new diagnostic algorithms. In this study, postaxial polydactyly was observed only in positive patients; however, no significant difference was detected, possibly due to the sample size.

Patient age at diagnosis is another factor to consider; for example, Campbell et al. (2018) reported that most of their patients were Caucasian and over 8 years old, whereas our cohort consisted primarily of Mestizo‐Mexican children aged 4–5 years. This difference may explain some clinical variations, as younger patients may not yet display the full phenotype due to age‐dependent features. Immune disorders, such as recurrent infections (sinusitis, otitis, pneumonia, bronchitis), are more frequently reported in children over 9 years, whereas atopic manifestations (food or drug allergies, eczema, asthma, rhinitis) are common before age 8 (Sullivan 2018). Similarly, intellectual disability often follows psychomotor delay and may go undetected in primary care, while hearing and vision problems tend to appear later, and psychiatric conditions typically emerge during or after adolescence (Wagner et al. 2017; Boot et al. 2023).

To assess whether deletion type influences clinical manifestations, the phenotype of patients with 3 Mb deletions was compared to those with smaller deletions. The absence of significant differences across phenotypes confirms that deletion size does not determine the clinical presentation (Campbell et al. 2018). Several theories have been proposed to explain phenotypic variability, including the role of miRNAs, both those encoded within the deleted region and others elsewhere in the genome that may act as modifiers. The role of *DGCR8* (OMIM *609030) has also been suggested since its haploinsufficiency in animal models leads to a 20%–70% reduction in miRNAs expressed in the prefrontal cortex, hippocampus, and heart (Bertini et al. 2017; Du et al. 2020; Colomer‐Boronat et al. 2025). It has also been proposed that the phenotypes associated with the deletion result not from individual haploinsufficiency, but from disruption of a multigenic interaction network. This is explained by the observation that genes within the region share molecular functions related to development, maturation, and cellular homeostasis. Furthermore, the LCR22A‐D region is located within a single, suggesting that deletions may affect normal chromatin interactions (Motahari et al. 2019; Zhao et al. 2020).

In patients who remain negative after FISH and MLPA evaluation, several possibilities should be considered. Differential diagnoses included Smith‐Lemli‐Opitz, CHARGE, and VATER syndromes, as well as other chromosomal microdeletions. If clinical suspicion for 22q11.2DS persists, targeted *TBX1* sequencing may detect pathogenic variants. Additionally, CMA, optical mapping, and whole genome sequencing can be employed to identify variants outside the 22q11.2 region that may explain the phenotype in negative patients.

## 5 Conclusion

Due to the large phenotypic variability reported in patients with 22q11.2DS, it was concluded that in FISH‐negative patients, a targeted search for atypical deletions is necessary. In positive subjects, characterization of the deletion by MLPA is important to define its size, breakpoints, and the genes involved, as well as to estimate frequencies of typical and atypical deletions. In addition, parents of patients with 22q11.2 deletions should be comprehensively assessed for the detection of somatic mosaicism. Finally, the evaluation of patients who remain negative should give way to other approaches, such as the analysis of gene interactions, epigenetics, and allelic variants outside the 22q11.2 region.

## Author Contributions

Thania Alejandra Aguayo‐Orozco: conceptualization, methodology, formal analysis, data curation, clinical data, writing – original draft. Horacio Rivera: conceptualization, methodology, supervision, writing – review and editing. Luis E. Figuera: methodology, clinical data, writing – review and editing. Eduardo Esparza‐García: methodology, clinical data, writing – review and editing. Francisco Javier Perea Díaz: methodology, supervision, writing – review and editing. Ana Rebeca Jaloma‐Cruz: methodology, supervision, writing – review and editing. Lourdes del Carmen Rizo‐de la Torre: methodology, formal analysis, supervision. Ma. Guadalupe Dominguez‐Quezada: conceptualization, methodology, funding acquisition, supervision, formal analysis, writing – review and editing. All authors approved the final version of the manuscript.

## Disclosure

No material was taken from other sources.

## Consent

All participants or their parents signed an informed consent form to participate in the study and granted permission for the publication of the findings.

## Conflicts of Interest

The authors declare no conflicts of interest.

## Supporting information


Data S1.

## Data Availability

The data that support the findings of this study are available from the corresponding author upon reasonable request.
